# Circulating Naïve Regulatory T Cell Subset Displaying Increased STAT5 Phosphorylation During Controlled Ovarian Hyperstimulation Is Associated with Clinical Pregnancy and Progesterone Levels

**DOI:** 10.3390/ijms27020922

**Published:** 2026-01-16

**Authors:** Ksenija Rakić, Aleš Goropevšek, Nejc Kozar, Borut Kovačič, Sara Čurič, Andreja Zakelšek, Evgenija Homšak, Milan Reljič

**Affiliations:** 1 Department of Reproductive Medicine and Gynaecological Endocrinology, Clinic for Gynaecology and Perinatology, University Medical Centre Maribor, Ljubljanska ulica 5, 2000 Maribor, Slovenia; ksenija.rakic@ukc-mb.si (K.R.); borut.kovacic@ukc-mb.si (B.K.); milan.reljic@ukc-mb.si (M.R.); 2 Faculty of Medicine, University of Maribor, Taborska ulica 8, 2000 Maribor, Slovenia; ales.goropevsek@ukc-mb.si (A.G.); evgenija.homsak@ukc-mb.si (E.H.); 3 Department of Laboratory Diagnostics, University Medical Centre Maribor, Ljubljanska ulica 5, 2000 Maribor, Slovenia; sara.curic@ukc-mb.si (S.Č.); andreja.zakelsek@ukc-mb.si (A.Z.)

**Keywords:** regulatory T cells, FOXP3, CD25, CD127, CXCR5, embryo implantation, in vitro fertilization

## Abstract

Regulatory T cells (Tregs), particularly their phenotypically distinct subpopulations, are critical for the establishment of maternal immune tolerance during embryo implantation. Despite advances in assisted reproductive technologies, implantation failure remains a frequent and often unexplained clinical challenge. Variations in Treg frequency and phenotype have been proposed to influence implantation success, particularly under differing hormonal conditions. This study aimed to investigate peripheral blood Treg levels and their subpopulations on the day of blastocyst transfer in both stimulated in vitro fertilization (IVF/ICSI) cycles involving controlled ovarian hyperstimulation (COH) and true natural cycles with frozen embryo transfer (FET), and to examine their associations with systemic hormone levels and anti-Müllerian hormone (AMH). A prospective observational study was conducted including women undergoing IVF/ICSI with fresh embryo transfer (ET) and women undergoing natural cycle FET. Peripheral blood samples were collected on the day of ET and analyzed using 13-colour flow cytometry, enabling detailed subdivision of Tregs into multiple subpopulations based on the expression of differentiation and chemokine markers, including CXCR5. In addition, because common γ-chain cytokines may influence pregnancy success by modulating the balance between suppressive Treg and non-Treg subsets, intracellular STAT5 signaling was assessed using phospho-specific flow cytometry. Serum estradiol, progesterone, FSH, LH, and AMH levels were measured in parallel. Significant differences were observed in Treg subpopulation distributions between women who conceived and those who did not. Higher frequencies of naïve CXCR5^−^ Tregs were associated with clinical pregnancy, independent of age, and correlated with serum progesterone levels. Moreover, both naïve Treg frequency and enhanced IL-7-dependent STAT5 signaling in naïve Tregs from women undergoing COH were associated with AMH levels, suggesting a link between ovarian reserve and Treg homeostasis mediated by signal transducer and activator of transcription 5 (STAT5) signaling. In conclusion, Treg subpopulations, particularly CXCR5^−^ naïve Tregs, appear to play a central role in implantation success following ET. Their distribution differs between stimulated and natural cycles and is influenced by systemic progesterone levels and STAT5 signaling. These findings suggest that peripheral Treg profiling may represent a potential biomarker of implantation competence and could inform personalized approaches in assisted reproduction.

## 1. Introduction

Even when embryos are chromosomally normal and of high morphological quality, a substantial proportion still fail to implant during in vitro fertilization with or without intracytoplasmic sperm injection (IVF/ICSI) treatment. The underlying causes of these implantation failures often remain unclear. Beyond embryonic factors, immunoregulatory mechanisms that suppress inflammation and promote maternal tolerance to paternal alloantigens have been recognized as critical contributors to successful implantation [1,2]. The FOXP3^+^ regulatory T cell (Treg) subset of CD4^+^ T cells plays a central role in maintaining peripheral immune tolerance in part by inhibiting the proliferation of other T cells, including effector CD4^+^ helper T cells [3]. Treg deficiency has been linked not only to various autoimmune diseases [4] but also to implantation failure and early pregnancy loss [5,6,7,8]. Although several classes of CD4^+^ T cells contribute to the suppression of immune responses, including Type 1 regulatory cells, CD4^+^FOXP3^+^ regulatory T cells (Tregs), distinguished also by high CD25 and low CD127 expression, are recognized as the principal subset within the female reproductive tract [9,10].

Zhou et al. reported in a prospective study that higher levels of peripheral blood Tregs were associated with increased conception rates in IVF procedures [11]. Moreover, dynamic changes in circulating Tregs appear to be clinically relevant: a transient elevation of peripheral Tregs on the day of embryo transfer has been associated with improved IVF outcomes [12]. A subsequent study, however, found no significant difference in the overall Treg-to-total CD4^+^ T cell ratio between women with successful and unsuccessful IVF outcomes. Notably, differences were observed in the distribution of Treg subpopulations: the proportion of naïve CD45RA^+^ Tregs was significantly lower in women who did not conceive [13]. It is important to note that these findings may have been influenced by the hormonal stimulation applied during IVF procedures. Previous studies have shown that Treg levels in peripheral blood vary during the menstrual cycle and correlate closely with those of estradiol [14]. In addition, estradiol levels vary widely in stimulated cycles and are on average several-fold higher than in the natural cycle. Supraphysiological levels of estradiol may also negatively affect implantation processes [15], potentially by altering immunoregulatory pathways, including the balance and functional properties of regulatory T cell subpopulations.

Due to recent advances in vitrification, frozen–thawed embryo transfer (FET) has become both increasingly common and clinically feasible [16]. In most IVF cycles, surplus embryos are vitrified for future use; in other cases, all embryos are frozen to prevent, for example, ovarian hyperstimulation syndrome or to allow for genetic testing. However, FET occurs in a markedly different hormonal environment compared to stimulated cycles [17], and emerging evidence suggests that these differences may influence maternal immune regulation, including autoimmune response [18]. Moreover, studies comparing stimulated and FET cycles have found significant differences in other peripheral CD4^+^ T cell populations [19], but not Treg subsets, defined by limited number of surface markers [13], underlining the need to use broader immunophenotyping panels/immune profiles when evaluating implantation outcomes [20].

The aim of our study was to characterize peripheral blood Treg levels and their subpopulations on the day of fresh ET in stimulated IVF cycles, as well as on the day of FET in natural cycles. We also investigated their relationship with serum hormone levels. Treg levels and their subpopulations were compared between successful and unsuccessful outcomes, and between natural and stimulated cycles. For a more detailed characterization of recently identified Treg subpopulations in blood samples from women undergoing IVF/ICSI, we employed immunophenotyping and 13-colour flow cytometry, enabling precise characterization of naïve, activated, and other specific subsets, including those expressing chemokine receptors such as CXCR5 [21]. As different factors, including cytokines, influence the success of pregnancy by increasing Treg cell number and activity [22], we assessed also their intracellular STAT signaling by using phosphospecific flow cytometry.

## 2. Results

### 2.1. Study Population

Included patients were ≤40 years of age on the day of ET (mean age: 32.6±3.8; range: 22–40), had a BMI < 35 kg/m^2^ (mean BMI: 22.7±3.5; range: 16–34) and AMH between 0.17–14.83 ng/L. More patient characteristics are presented in detail in (Table 1).

### 2.2. Correlation Between All Tregs, Defined as CD25^+^ CD127^lo/−^ Cells and Phenotypically Suppressive Subsets in Peripheral Blood from Women Undergoing IVF Procedures

**Figure 1:**
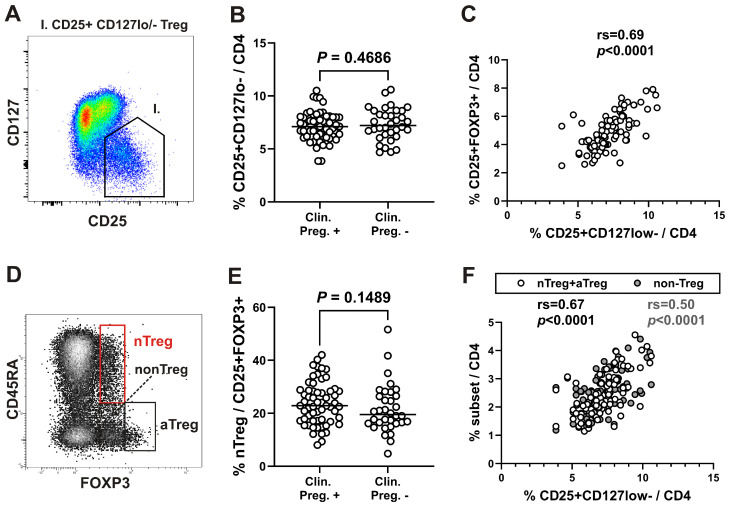
Correlation between all Tregs, defined as CD25^+^CD127^lo/−^ cells, and phenotypically suppressive naïve (nTreg) and activated (aTreg) subsets combined on the day of ET in peripheral blood from women undergoing IVF procedures. (**A**) Gating scheme for identification of all CD25^+^CD127^lo/−^ Treg cells among CD4^+^ T cells. (**B**) Scatter dot plot showing the percentage of CD25^+^CD127^lo/−^ cells among gated CD4^+^ T cells from women who became pregnant (clinical pregnancy +) compared to those who did not (clinical pregnancy −). (**C**) All CD25^+^FOXP3^+^ Treg cells were gated among CD4^+^ T cells (see Supplementary Figure S1). Correlation between the percentage of Tregs defined as CD25^+^FOXP3^+^ cells and CD25^+^CD127^lo/−^ cells among CD4^+^ T cells. (**D**) FOXP3^+^ cells among gated CD4^+^ T cells were subdivided into three fractions based on CD45RA and FOXP3 expression: (I) CD45RA^+^FOXP3^lo^ nTreg, (II) CD45RA^−^FOXP3^hi^ aTreg, and (III) CD45RA^−^FOXP3^lo^ non-Treg subsets, shown on a representative dot plot. (**E**) Percentage of nTreg subset among CD25^+^FOXP3^+^ cells from women who became pregnant compared to those who did not. (**F**) Correlation between CD25^+^CD127^lo/−^ Tregs and the sum of percentages of aTreg and nTreg subsets (white symbols) versus the non-Treg fraction (gray symbols) among CD4^+^ T cells. $r_{s}$, Spearman correlation coefficient.

We first assessed all Treg cells, defined as CD25^+^CD127^lo/−^ cells among CD4^+^ T cells (Figure 1A). The frequency of all CD25^+^CD127^lo/−^ cells was not significantly increased among CD4^+^ T cells in women who became pregnant compared to their nonpregnant counterpart (Figure 1B). When Treg cells were defined as CD25^+^FOXP3^+^ cells (Supplementary Figure S1), their percentage among CD4^+^ T cells was significantly correlated to percentage of CD25^+^CD127^lo/−^ cells among CD4^+^ T cells (Figure 1C). Next, Treg analysis was performed by using the strategy introduced by Miyara et al. [23] allowing functional delineation of FOXP3^hi^ expressing CD45RA^−^FOXP3^hi^ activated Treg (aTreg) subset and the two FOXP3^lo^ expressing subsets: CD45RA^+^FOXP3^lo^ resting or naïve Treg (nTreg), and CD45RA^−^FOXP3^lo^ (non-Treg) subset (Figure 1D). Although percentages of nTreg subset were higher in women who became pregnant compared to the women who did not, the difference was not statistically significant (Figure 1E). However, the percentage of CD25^+^CD127^lo/−^ Tregs among CD4^+^ T cells was more significantly correlated to the percentage of both Treg subsets combined (aTregs plus nTregs) than to the non-Treg fraction (Figure 1F).

### 2.3. Frequency of Circulating Naïve Treg Subset, Which Is Decreased in Women Who Did Not Conceive After COH, Is Associated with AMH Levels

**Figure 2:**
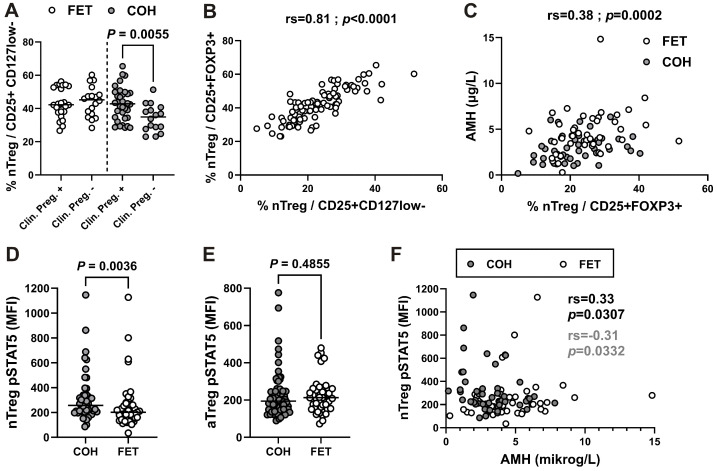
Frequency of circulating naïve Treg subset, which is decreased in women who did not conceive after COH, as well as STAT5 phosphorylation levels in the same subset, are associated with AMH levels. (**A**) Scatter dot plot showing the percentage of nTreg subset among gated CD25^+^CD127^lo/−^CD4^+^ T cells from women who became pregnant compared to those who did not in the group undergoing COH (gray symbols) or FET in a natural cycle (white symbols). (**B**) Correlation between the percentage of nTreg, defined as CD45RA^+^CD25^lo^ cells among CD25^+^CD127^lo/−^ Tregs, and the percentage of nTreg, defined as CD45RA^+^FOXP3^lo^ cells among CD25^+^FOXP3^+^ Tregs. (**C**) Correlation between the percentage of nTregs among CD25^+^FOXP3^+^ Tregs and AMH levels from women undergoing COH (gray symbols) or FET in a natural cycle (white symbols). (**D**,**E**) Basal pSTAT5 levels (MFI) in nTreg and aTreg subsets, respectively, from women undergoing COH or FET in a natural cycle. (**F**) Correlation between pSTAT5 levels (MFI) in the nTreg subset and AMH levels from women undergoing COH (gray symbols) or FET in a natural cycle (white symbols).

Next, we compared FOXP3^+^ subsets in the more homogeneous study groups that underwent either COH or FET in a true natural cycle on the day of optimal blastocyst transfer. In the group undergoing COH, women who achieved pregnancy had a significantly higher percentage of CD45RA^+^ nTreg cells within CD25^+^CD127^lo/−^ Treg cells (Figure 2A), which was well correlated with the percentage of CD45RA^+^ nTreg cells within CD25^+^FOXP3^+^ Treg cells (Figure 2B). Furthermore, when Treg subsets were analyzed in relation to AMH levels, only the nTreg subset showed a significant correlation with AMH in both patients undergoing FET and those in the COH group (Figure 2C). These results suggest that the nTreg subset, which was significantly increased in women who achieved pregnancy after COH but not after FET, may be associated with ovarian reserve in both groups.

### 2.4. Increased STAT5 Phosphorylation Levels in the nTreg Subset in Women Undergoing COH Are Associated with Lower AMH Levels

IL-7-induced STAT5 signaling has previously been implicated in the homeostasis of the naïve Treg subset [24]. Therefore, we next compared levels of basal STAT5 phosphorylation (pSTAT5) in suppressive nTreg and aTreg subsets. We found that pSTAT5 levels in nTregs (Figure 2D), but not in aTregs (Figure 2E), were significantly increased in women undergoing COH compared with women undergoing FET. In addition, significant negative and positive correlations were observed between AMH levels and nTreg pSTAT5 levels in women undergoing COH and FET, respectively (Figure 2F). Finally, pSTAT5 levels were reduced by incubation with neutralizing anti-IL-7, but not anti-IL-2, antibodies, suggesting that the increased basal STAT5 phosphorylation in nTregs from women undergoing COH is IL-7 dependent (Supplementary Figure S2).

### 2.5. Unsupervised Cell Clustering Analyses Showed Decreased Naïve Treg Subset Lacking CXCR5 Expression in Women Who Did Not Conceive

**Figure 3:**
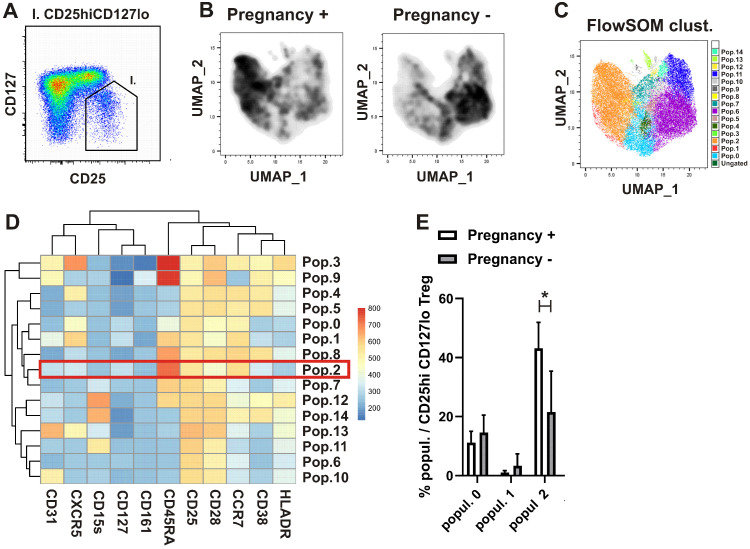
Increased nTreg-like population lacking CXCR5 expression identified via unsupervised cell clustering analyses in women who conceived after undergoing COH. (**A**) Cytometry data acquired from blood samples from women undergoing COH were gated in FlowJo software as shown, and equal numbers of CD25^+^CD127^lo/−^ Treg cells (gating hierachy shown on Supplementary Figure S5) from women who conceived or did not were included in the analysis. (**B**) Differences in the distribution of clustered populations as evidenced by the UMAP analysis. (**C**) A total of 15 phenotypically distinct populations within Treg cells identified using the FlowSOM clustering lalgorithm are shown. (**D**) Populations were identified and clustered by the lalgorithm according to their expression of surface markers CD25 (IL-2Rα), CD127 (IL-7Rα), CCR7, CXCR5, CD28, CD38, CD161, CD31, CD45RA, HLA-DR, and CD15s. (**E**) Percentages of population 2, identified using unsupervised clustering, among CD25^+^CD127^lo/−^ Treg cells from women who conceived or did not after undergoing COH. * p<0.05.

Standard gating is limited to the analysis of only two markers at a time in a flow cytometry plot. In addition, marker expression levels are often not taken into account, favoring a simplified distinction between “positive” and “negative” populations. As an alternative approach, dimensionality reduction analyses were performed using the UMAP and FlowSOM cell clustering algorithms in *FlowJo* v10.8. UMAP enables clustering of cells based on the expression levels of multiple markers in a two-dimensional (2D) space, while FlowSOM identifies phenotypically distinct populations that can be visualized in the UMAP 2D output. Accordingly, thirteen-color flow cytometry data were analyzed using UMAP and FlowSOM. By combining these tools, we analyzed the phenotypes of cell populations in samples from women undergoing COH who became pregnant compared with those who did not, within Treg cells defined as CD25^+^CD127^lo/−^ CD4^+^ T cells (Figure 3A). Equal numbers of CD25^+^CD127^lo/−^ CD4^+^ T cells were included in the analysis and clustered according to their expression levels of the surface markers CD25 (IL-2Rα), CD127 (IL-7Rα), CCR7, CXCR5, CD28, CD38, CD161, CD31, CD45RA, HLA-DR, and CD15s. FlowSOM identified 15 phenotypically distinct populations within the Treg compartment (Figure 3B). UMAP analysis revealed differences in the distribution of clustered populations, with some clusters being over- or under-represented in women who became pregnant (Figure 3C), while remaining grouped according to their expression levels of the analyzed markers (Figure 3D). The most prevalent population that was increased in women who became pregnant exhibited an nTreg-like phenotype lacking CXCR5 expression (Figure 3E).

### 2.6. CXCR5-Negative Naïve Treg Subset Is Associated with Clinical Pregnancy After COH, Regardless of the Woman’s Age, and Correlates with Progesterone Levels

**Figure 4:**
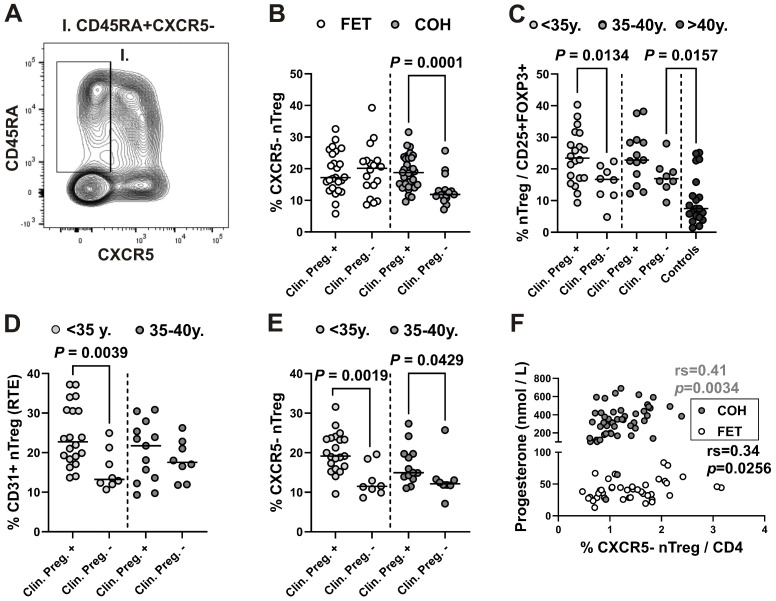
Increased frequency of CXCR5^−^ nTreg subset is associated with clinical pregnancy after undergoing COH, regardless of the women’s age, and correlates with progesterone levels. (**A**) Gating scheme for identification of the CXCR5^−^CD45RA^+^ nTreg subset among CD25^+^CD127^lo/−^ Treg cells (see also Supplementary Figure S6). (**B**) Scatter dot plot showing the percentage of CXCR5^−^CD45RA^+^ nTreg subset among gated CD25^+^CD127^lo/−^CD4^+^ T cells from women who became pregnant compared to those who did not in the COH group (gray symbols) or FET in a natural cycle (white symbols). (**C**) Percentage of nTreg subset among gated CD25^+^FOXP3^+^ Tregs from women undergoing COH who became pregnant compared to those who did not, in women younger than 35 years (white symbols), older than 35 and younger than 40 years (gray symbols), and in healthy control women older than 40 years (black symbols). (**D**) Percentage of CD31^+^ nTreg subset among gated CD25^+^FOXP3^+^ Tregs from women undergoing COH who became pregnant compared to those who did not, in women younger than 35 years (white symbols) and in women aged 35–40 years (gray symbols). (**E**) Percentage of CXCR5^−^ nTreg subset among gated CD25^+^FOXP3^+^ Tregs from women undergoing COH who became pregnant compared to those who did not, in women younger than 35 years (white symbols) and in women aged 35–40 years (gray symbols). (**F**) Correlation between the percentage of CXCR5^−^ nTreg subset among CD4^+^ T cells and progesterone levels from women undergoing COH (gray symbols) or FET in a natural cycle (white symbols).

When we related the results of unsupervised clustering to those obtained by standard gating (Figure 4A), we again observed that the percentage of CXCR5^−^ nTreg cells within CD25^+^CD127^lo/−^ Treg cells was significantly higher in pregnant women (Figure 4B). When Treg frequencies in women undergoing COH were compared across two age groups (<35 years and 35–40 years) and with twenty healthy adult females older than 40 years (mean age 51.2 years; range 40.8–63.3 years), nTreg frequency was significantly lower in females older than 40 years, even when compared with women aged 35–40 years who did not conceive after COH. Moreover, nTreg frequency was significantly higher only in women younger than 35 years who became pregnant compared with women in the same age group who did not conceive, whereas in women aged 35–40 years the difference was not significant (Figure 4C). Although women in our control group of women aged 40+ had no history of allergies, acute infections, autoimmune disorders or immunosuppressive medicines, age is a powerful confounding variable for immune function. However, changes in the composition of the total Treg pool with distinct Treg subsets according to age were shown before, including the decrease in percentage of naïve CD45RA^+^-Tregs with age [13].

Naïve T cells that have recently emigrated from the thymus (recent thymus emigrants [RTEs]) have well-established properties, including a decline with increasing age. Of note, the onset of pregnancy has recently been associated with a marked decrease in RTE-Tregs [25]. Although the reference method for RTE measurement is quantification of T cell receptor excision circles in peripheral blood mononuclear cells, Kimmig et al. [26] demonstrated that CD31 expression on naïve CD4^+^ T cells identifies a population that overlaps with RTEs. When we analyzed CD31 expression on nTregs, it was significantly inversely correlated with the age of women undergoing IVF procedures (Supplementary Figure S3). In addition, as with total nTreg cells, RTE-nTreg frequencies were significantly higher only in women younger than 35 years who became pregnant compared with those in the same age group who did not, whereas in the 35–40–year age group the difference was not significant (Figure 4D). In contrast, CXCR5^−^ nTreg frequency was significantly higher in women from both age groups undergoing COH who became pregnant compared with women who did not (Figure 4E).

As the results may also have been influenced by the hormonal stimulation used in IVF procedures, we investigated the relationship between serum hormone levels and Treg levels. As expected, progesterone levels were significantly higher in women undergoing COH compared to those undergoing FET (Supplementary Figure S4). No significant differences in progesterone levels were observed between women who conceived and those who did not in either the COH or FET groups (Supplementary Figure S4B). However, progesterone levels were significantly correlated with the percentage of CXCR5^−^ nTreg cells among CD4^+^ T cells in both women undergoing COH and those undergoing FET in a natural cycle (Figure 4F).

To further account for potential confounding, the association between the proportion of CXCR5^−^ nTreg cells and pregnancy outcome was additionally evaluated using multivariable logistic regression adjusted for female age, AMH, and BMI (Supplementary Table S3). In the multivariable analysis, only the proportion of CXCR5^−^ nTreg cells was a significant predictor (p<0.05), whereas the other covariates did not reach statistical significance. Bonferroni correction was applied for multiple testing (Supplementary Table S4).

## 3. Discussion

Research on mouse models has demonstrated that during each reproductive cycle, the pool of Treg cells prior to potential embryo implantation, during the so-called implantation window of the embryo, increases. These cells are recruited from peripheral blood into the tissues of the reproductive tract and are subsequently activated and differentiated under the influence of sex hormones and factors present in seminal fluid [27,28,29,30]. Similar mechanisms of recruitment and amplification of maternal Treg cells are also likely to be essential in humans [14,31].

Therefore, based on previous reports showing that higher total regulatory T cell levels in peripheral blood are associated with increased conception rates in IVF procedures [11], we first assessed the frequency of CD25^+^CD127^lo/−^ cells. The proportion of these cells among CD4^+^ T cells did not differ significantly between women who achieved pregnancy and those who did not. The percentage of CD25^+^CD127^lo/−^ cells within the CD4^+^ T cell compartment was strongly correlated with the proportion of CD25^+^FOXP3^+^ cells, which are commonly regarded as representing the “classical” regulatory T cell phenotype. Moreover, CD25^+^CD127^lo/−^ Tregs showed a stronger correlation with the combined frequency of activated and naïve Treg subsets (aTregs and nTregs) than with the non-Treg fraction. This finding supports the notion that the majority of phenotypically suppressive cells reside within the nTreg and aTreg subsets rather than within the non-Treg compartment, consistent with the established association between Treg suppressive function and high CD25 and low CD127 expression [32,33].

Functional delineation of FOXP3^+^ cell subsets based on CD45RA expression and FOXP3 levels revealed that the percentages of phenotypically naïve CD45RA^+^FOXP3^+^ nTregs among all CD25^+^FOXP3^+^ Tregs were lower in women who did not become pregnant compared to those who did, although the difference was not significant. However, when we compared FOXP3^+^ subsets in more homogeneous study groups, specifically in the cohort that underwent COH, the percentages of Treg subsets within CD25^+^FOXP3^+^ Treg cells were significantly different in women who achieved pregnancy.

Results of previous studies on Treg subsets showed a significantly reduced percentage of naïve CD45RA^+^ Tregs in women who did not conceive after IVF procedures, even in patients who were treated uniformly with COH [13]. Consistent with these findings, in our cohort of women undergoing COH, those who did not achieve pregnancy had a significantly lower percentage of CD45RA^+^ nTreg cells within CD25^+^CD127^lo/−^ Treg cells, which was well correlated with the percentage of CD45RA^+^ nTreg cells within CD25^+^FOXP3^+^ Treg cells.

Taken together, these results suggest that, at least in women undergoing COH, a sufficient systemic supply of circulating naïve Treg cells is most likely required for successful embryo implantation. However, results of a recent study demonstrated that non-naïve CD45RA^−^ Tregs could be used as immunologic markers of reproductive aging, and the authors also reported a significant negative correlation between activated HLA-DR^+^CD45RA^−^ Tregs and AMH levels [34]. In contrast, we found that only the nTreg subset was significantly correlated with AMH levels in patients undergoing vitrified-warmed blastocyst transfer (FET) as well as in those undergoing COH. These findings suggest that the nTreg subset, which was significantly increased in women who achieved pregnancy after COH, may be related to ovarian reserve in both the COH and FET groups.

In addition, we found significantly increased levels of IL-7-dependent STAT5 phosphorylation in the same nTreg subset, but not in aTregs, in women undergoing COH compared to women undergoing FET in a natural cycle. IL-2-dependent activation and phosphorylation of STAT5 are essential for the development and suppressive function of Tregs, as this pathway also regulates their key transcription factor, FOXP3 [35]. Recent studies [24] have shown that another homeostatic cytokine, IL-7, is involved in the long-term thymus-independent survival of phenotypically naïve/resting CD45RA^+^ nTregs. Consistent with these findings, in our cohort of women undergoing COH, the nTreg subset displayed significantly higher STAT5 phosphorylation levels. Moreover, the significant negative correlation between AMH levels and nTreg pSTAT5 levels in women undergoing COH suggests that both nTreg frequency and IL-7-dependent STAT5 signaling within this subset may be related to stimulation capacity dependent on ovarian reserve.

IL-7 is one of the prototypical factors secreted by oocytes, and its secretion increases three- to fivefold as the oocyte matures. IL-7 secretion is barely detectable in germinal vesicle (GV) oocytes and increases significantly when oocytes reach the metaphase II (MII) stage. A similar pattern is observed in the follicular fluid of ovarian follicles before and after hCG stimulation in vivo. Notably, IL-7 secretion becomes undetectable after fertilization, which is further supported by the large fluctuations in interleukin concentrations in the environment surrounding the cumulus–oocyte complex [36]. Of note, higher systemic IL-7 levels in plasma from women undergoing COH compared with those undergoing FET in a natural cycle were recently reported [37]. Consistent with this, IL-7-dependent pSTAT5 levels in the nTreg subset were significantly higher in women undergoing COH than in those undergoing FET in a natural cycle.

In a recent study of peripheral blood cells in the mid-luteal phase of the menstrual cycle involving 27 female subjects with early pregnancy failure (EPF), who had clinical features of recurrent miscarriage, recurrent implantation failure, or a combination of both, the authors reported a significant decrease in the CD45RA^+^CCR7^+^ naïve Treg subpopulation compared with controls [38]. Among 15 phenotypically distinct populations of Treg cells identified by 13-colour immunophenotyping and unsupervised FlowSOM clustering, the most prevalent population that was increased in our women who achieved pregnancy displayed a CD45RA^+^CCR7^+^ naïve nTreg-like phenotype, which, however, lacked CXCR5 expression. The latter marker defines a distinct subpopulation of T follicular regulatory cells (Tfr) involved in the suppression of humoral immune responses, including within germinal centers of lymph nodes [39,40]. In the aforementioned study, in addition to the significant decrease in the CD45RA^+^CCR7^+^ naïve Treg subpopulation, dysregulation of gene expression within Treg cells of EPF subjects was also described. Although CXCR5 and PDCD1 (encoding PD-1), both of which are expressed by Tfr cells, were found to be upregulated—indicating increased Tfr differentiation—other genes normally associated with a Tfr signature, such as BCL6 and ICOS, were not differentially expressed [38]. In line with these observations, using newer bioinformatics tools with confirmation by conventional gating, we found significantly lower proportions of nTreg cells lacking CXCR5 expression in women who did not become pregnant. Therefore, our findings suggest that the composition of distinct populations within the naïve Treg compartment may influence a woman’s chance of becoming pregnant, particularly nTregs lacking expression of the chemokine receptor CXCR5.

From the age of 40 years, a preferential conversion of naïve CD45RA^+^ Tregs into memory-like CD45RA^−^HLA-DR^−^ Tregs has been reported in a recent study [13]. It was therefore suggested that, in addition to ovarian aging, an age-related shift in the composition of the total Treg pool may also contribute to the loss of female fertility. Consistent with this, nTreg frequency was significantly lower in women older than 40 years, even when compared with our group of women aged 35–40 years who did not conceive after undergoing COH. However, irrespective of age, the percentage of naïve CD45RA^+^ Tregs was found in the aforementioned study to be significantly decreased in patients who did not become pregnant after IVF/ICSI treatment [13]. In contrast, in our cohort, nTregs were significantly lower only in women younger than 35 years who did not become pregnant compared with those in the same age group who did, whereas in the group of women aged 35–40 years the difference was not significant. Insufficient thymic generation of new Treg cells may also contribute to a depleted naïve Treg cell pool with advancing age in women undergoing IVF procedures. Indeed, when we quantified recent thymic emigrants (RTEs) using CD31 expression on naïve Tcon and Treg cells, their frequency was significantly inversely correlated with age.

In addition, as observed for total nTregs, significantly higher frequencies of RTE-nTregs were found only in women younger than 35 years who became pregnant compared with women in the same age group who did not, whereas in the group of women aged 35–40 years the difference was not significant. By contrast, the frequency of CXCR5^−^ nTregs was significantly higher in women from both age groups undergoing COH who became pregnant compared with those who did not. Therefore, our results suggest that the CXCR5^−^ nTreg subset, which was the most significantly increased among all subsets in women who conceived after COH, may be associated with the success of this IVF procedure regardless of age.

Furthermore, the percentage of CXCR5^−^ nTregs among CD4^+^ T cells in both women undergoing COH and those undergoing FET in a natural cycle was significantly correlated with progesterone levels. Results from a recent mouse study demonstrated that Treg cells are causal mediators of adverse pregnancy outcomes following impaired progesterone signaling in early pregnancy, and that restoration of Treg cells was sufficient to alleviate the effects of insufficient progesterone signaling [26]. In another study, conditional loss of the canonical nuclear progesterone receptor in maternal FOXP3^+^ regulatory T cells was shown to blunt their proliferation and accumulation, which was associated with fetal wastage and decidual infiltration by activated CD8^+^ T cells [41]. Progesterone has also been shown to drive the differentiation of human cord blood naïve T cells into Tregs [42]. Notably, progesterone enhanced STAT5 activation in response to IL-2, consistent with its selective role in promoting Treg generation over pro-inflammatory Th17 cells [42].

On the other hand, elevated systemic levels of IL-7, which may depend on ovarian stimulation and support the nTreg subpopulation [24], could contribute to cyclical shifts between different Treg subpopulations [43].

This study has several limitations. First, it was conducted at a single tertiary center and included a predominantly White patient population from a single geographic region, which may limit the generalizability of the findings to other populations and clinical settings. Second, although only morphologically optimal blastocysts were transferred, preimplantation genetic testing for aneuploidy (PGT-A) was not performed; therefore, embryo aneuploidy cannot be excluded as a contributor to implantation failure independent of the immune parameters assessed. Third, immune profiling was performed in peripheral blood at a single time point, on the day of ET. As a result, peripheral immune signatures may not fully reflect the local immune environment within the endometrium or decidua. In addition, the cross-sectional design precludes assessment of temporal dynamics in immune regulation during the peri-implantation window, which may be critical for successful implantation. Finally, the relatively modest sample size for certain immune subpopulations may have limited statistical power to detect small but biologically relevant effects.

## 4. Materials and Methods

### 4.1. Study Population

A total of 50 patients undergoing controlled ovarian hyperstimulation (COH) with a short GnRH antagonist protocol and 44 patients undergoing FET in a true natural cycle were included in the analysis. Only the first and second consecutive single optimal blastocyst transfers (grade ≥ 4, 5AA according to the Gardner and Schoolcraft criteria [44]) in patients aged ≤40 years were prospectively included. The cohort was selected to represent good-prognosis patients undergoing standard infertility treatment in a single, tertiary center. The study was approved by the institutional ethics committee (approval no. UKC-MB-KME-4/20), and written informed consent was obtained from all participants. The study was conducted between March 2020 and October 2024. Patients with uterine anomalies (e.g., polyps, fibroids, or a uterine septum), adenomyosis, or an endometrial thickness < 7 mm were excluded.

### 4.2. Samples Collection and Hormonal Analysis

In addition to flow cytometric analyses, serum estradiol and progesterone levels were measured on the morning of ET. Basal levels of FSH, LH, and AMH were assessed using samples collected on cycle days 2–5, approximately 2–3 months prior to IVF treatment.

Serum AMH concentrations were measured using the Elecsys AMH automated electrochemiluminescence immunoassay (ECLIA) on a Cobas 601 analyzer (Roche Diagnostics, Mannheim, Germany). The reference range applied for women of reproductive age was 0.26–12.0 ng/L.

Serum estradiol, progesterone, LH, and FSH levels were determined using a chemiluminescent microparticle immunoassay (CMIA) on the Alinity i automated immunoassay analyzer (Abbott Laboratories, North Chicago, IL, USA), employing anti-analyte-coated paramagnetic microparticles and acridinium-labeled anti-analyte conjugates.

### 4.3. COH and FET Procedures

Ovarian stimulation was performed using a fixed GnRH antagonist protocol with recombinant FSH or human menopausal gonadotropin (hMG), and ovulation was triggered with recombinant human chorionic gonadotropin (hCG). IVF/ICSI was subsequently performed, followed by fresh transfer of a single optimal blastocyst (grade ≥ 4, 5AA).

FET cycles involving day 5 or day 6 optimal blastocysts were conducted in true natural cycles (tNC) using urinary luteinizing hormone (LH) detection, with all transfers performed on days LH+5, LH+6, or LH+7. Luteal phase support with progesterone (Utrogestan (Cyndea Pharma, Ólvega, Spain), 200 mg twice daily) was initiated on the day of FET and continued for two weeks. The ovarian stimulation and FET protocols have been described in detail in our previous study [45].

The primary outcome was clinical pregnancy, defined as ultrasonographic confirmation of an intrauterine gestational sac with a detectable fetal heartbeat.

### 4.4. Preparation of EDTA-Anticulated/Whole Blood Samples for Analysis of Basal STAT5 Signaling/Phosphorylation

Basal activation/phosphorylation of STAT5 immediately ex vivo without prior stimulation was studied in whole blood/EDTA-anticoagulated samples. Preparation of samples included fixation to stop phosphorylation and lysing of erythrocytes with BD Phosflow Lyse/Fix Buffer (BD Biosciences, San Jose, CA, USA). 2 mL of the mentioned buffer were used at 1:5 dilution for fixation of samples (100 μL), which were subsequently incubated for 10 min in tubes in a prewarmed (37 °C) water bath. In selected experiments, whole blood samples were incubated with neutralizing antibodies anti-IL-2 (2 μg mL^−1^; clone MQ1-17H12; BD Biosciences) and anti-IL-7 (1 μg mL^−1^; clone BVD10-40F6; BD Biosciences) for 30 min at 37 °C before fixation. Subsequent preparation of samples included centrifugation for 7 min (300× *g*), and permeabilization of cells with BD Perm Buffer III (BD Biosciences). Permeabilization of cells with the mentioned buffer, which is based on methanol, was performed by first slowly adding cold buffer while vortexing and later incubation on ice for 30 min. Final steps of sample preparation before the staining with antibodies included washing cells (2×) with phosphate-buffered saline (2 mL of PBS and centrifugation at 300× *g*).

### 4.5. Combined Surface/Intracellular Staining for Analysis on a Flow Cytometer

Combined staining of surface/intracellular epitopes was performed in PBS (phosphate buffered saline) buffer/with 2% FBS (fetal bovine serum) (Thermo Fisher Scientific, Waltham, MA, USA). Antibodies used for staining samples (100 µL) for 30 min at room temperature are shown in Supplementary Table S1. Finally, after washing cells (1×) with buffer (2 mL of PBS/with 2% FBS and centrifugation at 300× *g*) samples were acquired on FACSymphony A3 flow cytometer (BD Biosciences, Franklin Lakes, NJ, USA). Analysis (including measurement of pSTAT5 MFI-median fluorescence intensity) was performed using FACSDiva software version 9.1 (BD Biosciences). FlowJo software version 10.8.1 (TreeStar, Ashland, OR, USA, now part of BD Biosciences) was also used for flow cytometry analysis.

### 4.6. Surface Immunophenotyping Using a 13-Colour Panel

When Treg subpopulations were determined on the basis of surface antigens in a 13-colour panel, the following antibodies (Thermo Fisher Scientific, Waltham, MA, USA) were used simultaneously: anti-CXCR5 (CD185)-BB515 (3 µL, clone RF8B2), anti-CD161 PE (10 µL, clone DX12), anti-CCR7 (CD197) PE-CF594 (3 µL, clone 2-L1-A), anti-HLA-DR PerCP-Cy5.5 (10 µL, clone L243), anti-CD45RA-PECy7 (0.5 µL, clone HI100), anti-CD127-Alexafluor647 (10 µL, clone HIL-7R-M21), anti-CD38-APC-H7 (3 µL, clone HB7), anti-CD25-BV421 (3 µL, clone 2A3), anti-CD15s-BV510 (3 µL, clone CSLEX1), anti-CD31-BV605 (3 µL, clone WM59), anti-CD28-BV711 (3 µL, clone CD28).

Aliquots of whole blood (100 µL) from EDTA tubes were treated with 2 mL of 10 × diluted FACSlyse erythrocyte lysis buffer (BD Biosciences) after 15 min of labelling with the described monoclonal antibodies in the dark. The samples were then centrifuged at 300× *g* for 5 min, washed with 2 mL Stain Buffer BSA (BD Pharmingen/BD Biosciences). Finally, the samples were centrifuged again at 300× *g* for 5 min and 500 µL of the same Stain Buffer was added prior to analysis on FACSymphony A3 Flow Cytometer.

### 4.7. Unsupervised Analysis by Flow Cytometry

Cytometry data acquired from blood samples were first analyzed and gated in FlowJo software to remove debris and doublets based on FSC/SSC discrimination. Subsequently, CD3^+^CD4^+^CD25^+^CD127^lo/−^ Treg cells were gated, and an equal number of events from each sample were pooled to represent the whole sample using the FlowJo DownSample lalgorithm. The UMAP (Uniform Manifold Approximation and Projection) dimensionality reduction lalgorithm was applied, which operates similarly to tSNE (t-distributed stochastic neighbor embedding). However, UMAP has no computational restrictions on embedding dimensionality and more effectively preserves the global structure of the data. The number of metaclusters was determined by initially running the X-shift plugin/lalgorithm, followed by final hierarchical clustering using the FlowSOM lalgorithm to assign all events into a defined number of metaclusters.

### 4.8. Statistical Analysis

Statistical analysis was performed using GraphPad Prism version 10 for Windows (GraphPad Software, San Diego, CA, USA). Non-parametric tests were used for between-group comparisons, while the Wilcoxon matched-pairs signed-rank test was applied for within-group comparisons. Associations between variables were assessed using Spearman’s rank correlation coefficient. *p* values <0.05 were considered statistically significant. Bonferroni correction was applied for multiple testing. Post hoc power analysis was performed for the primary regulatory T cell subpopulation endpoints using a two-sample comparison of continuous variables (Supplementary Table S2).

## 5. Conclusions

Collectively, our results show that both nTreg frequency and IL-7-dependent STAT5 signaling—possibly supporting nTreg differentiation—were associated with lower AMH levels and were significantly increased in women undergoing COH, suggesting a link with ovarian reserve-dependent stimulation. In addition, our data support the interpretation that although thymic generation of new RTE-Tregs decreases with age in women undergoing IVF procedures, progesterone may support differentiation of the specific CXCR5- nTreg subset, which was associated with successful COH outcomes regardless of the women’s age.

## Figures and Tables

**Table 1:** Stratified comparison by cycle type (COH and FET): median [IQR] within outcome groups and Wilcoxon *p*-values.

	COH Cycles			FET Cycles		
Variable	Pregnant	Not-Pregnant	*p*	Pregnant	Not-Pregnant	*p*
**Demographics and basal hormonal levels**						
Age, years	33.0 [30.0–36.0]	35.0 [31.0–36.0]	0.489	32.0 [29.0–34.0]	31.5 [30.0–33.0]	0.856
BMI, kg/m^2^	22.0 [20.0–25.0]	22.0 [21.0–25.0]	0.623	23.0 [20.0–25.0]	22.0 [20.0–23.0]	0.402
AMH, ng/mL	2.86 [2.11–3.84]	2.95 [1.78–4.25]	0.755	4.68 [3.66–6.18]	3.81 [2.32–5.15]	0.077
FSH, IU/L	5.90 [4.55–7.35]	6.00 [5.40–6.20]	0.909	5.52 [4.90–6.60]	5.80 [4.90–6.75]	0.887
LH, IU/L	3.70 [2.45–4.85]	4.20 [3.20–5.70]	0.240	4.15 [3.00–5.10]	5.15 [3.20–6.15]	0.233
**Measurements on ET day**						
Endometrial thickness, mm	10.0 [8.0–12.7]	10.0 [9.0–12.0]	0.724	8.5 [8.0–10.0]	8.0 [7.0–9.0]	0.021
Estradiol (E2), ng/mL	4.14 [2.79–5.41]	3.16 [1.81–5.80]	0.385	0.41 [0.32–0.53]	0.45 [0.33–0.50]	0.982
Progesterone (P4), ng/mL	346.8 [271.0–447.4]	296.8 [121.4–420.6]	0.106	38.1 [29.2–45.3]	38.1 [24.6–58.0]	0.864
**Pregnancy outcome, n (%)**						
Pregnant	35 (70.0%)			28 (60.9%)		
Not-pregnant	15 (30.0%)			18 (39.1%)		

Values are presented as median [interquartile range] or counts (%). *p*-values were calculated using the Wilcoxon rank-sum test within each cycle type.

## Data Availability

Data are available upon reasonable request from the corresponding author.

## Supplementary Materials

The following supporting information can be downloaded at: https://www.mdpi.com/article/10.3390/ijms27020922/s1.
