# Supplementary material for: Circulating Naïve Regulatory T Cell Subset Displaying Increased STAT5 Phosphorylation During Controlled Ovarian Hyperstimulation Is Associated with Clinical Pregnancy and Progesterone Levels
Source: Int J Mol Sci. 2026 Jan 16;27(2):922. doi: 10.3390/ijms27020922 (PMC12841674; doi:10.3390/ijms27020922)
Supplement: Supplementary file 1 [file ijms-27-00922-s001.zip › ijms-4025173-supplementary.pdf]

Supplementary Materials

Supplementary Table 1: Antibodies used for basal STAT5 signaling analysis

Antibody	Fluorochrome	Volume per sample	Clone
anti-CD45	BV786 or APC-Cy7	1 μL	HI30
anti-CD3	BV786	5 μL	2D1
pSTAT5 (Y694)	Alexa647	1 μL	SK7
anti-CD4	BV750	10 μL	47
anti-CD25	BV421	2 μL	SK3
anti-CD45RA	PE-Cy7	3 μL	2A3
anti-Ki67	BV650 or PE	0.5 μL	HI100
anti-FOXP3	Alexa488 or PE	3 μL, 10 μL, 10 μL	B56, B56, 259D/C7, 259D/C7

All antibodies were purchased from BD Biosciences.

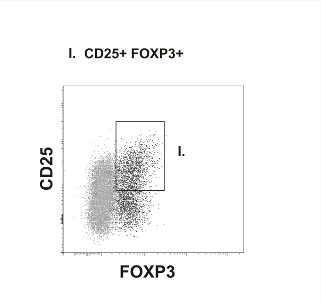

Supplementary Figure 1: Gating for identification of CD25+ FOXP3+ Treg among CD4+ T cells

Supplementary Table 2: Supplementary Table S2. Post hoc power analysis for selected Treg subpopulations (Pregnant vs. Not-pregnant).

Variable	n_{P}	n_{NP}	Cohen's d	Power
pSTAT5 nTreg	63	33	0.12	0.09
aTreg	63	33	-0.61	0.81
CD15s+ aTreg	61	33	-0.11	0.08
Ki67+aTreg	63	33	-0.40	0.45
CXCR5+ nTreg	62	33	-0.33	0.32
CXCR5- nTreg med CD4	62	33	0.40	0.45
RTE nTreg med Treg	61	33	0.45	0.54

Supplementary Table 3: Multivariable logistic regression analysis evaluating the association between the proportion of $CXCR5^{-}$ nTreg (median proportion within total Treg) and the probability of pregnancy in COH cycles. The model was adjusted for female age, AMH, and BMI. Results are presented as regression coefficients with standard errors in parentheses.

	Dependent variable:
	Pregnancy
CXCR5neg_nTreg	0.214**
	(0.088)
Age	−0.046
	(0.100)
AMH	0.067
	(0.233)
BMI	0.007
	(0.090)
Constant	−1.350
	(4.090)
Observations	45
Log Likelihood	−23.021
Akaike Inf. Crit.	56.042
Note:	*p<0.1; **p<0.05; ***p<0.01

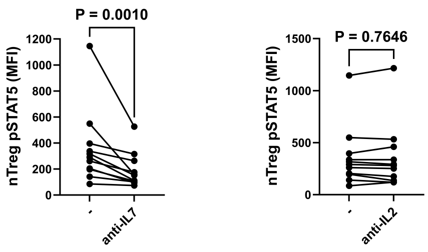

Supplementary Figure 2: pSTAT5 levels (MFI) in nTreg from women undergoing COH: basal pSTAT5 levels are compared to pSTAT5 levels after incubation with neutralizing anti-IL-2 and anti-IL-7 antibodies

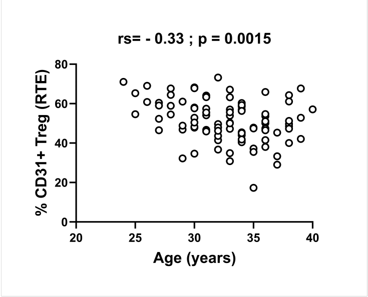

Supplementary Figure 3: Corelation between percentage of CD31+ cells among gated nTreg cells and age

Supplementary Table 4: Univariate analysis of selected Treg subpopulations with Bonferroni correction for multiple testing (six comparisons).

Treg subpopulation	p value	Bonferroni-adjusted p
$CD25^+CD127^{lo/-}$ Treg among CD4	0.4686	1.0000
nTreg among $FOXP3^+CD25^+$ Treg	0.1489	0.8934
nTreg pSTAT5 (MFI)	0.0036	0.0216
aTreg pSTAT5 (MFI)	0.4855	1.0000
nTreg among $CD25^+CD127^{lo/-}$ Treg (COH)	0.0055	0.0330
$CXCR5^-$ nTreg among Treg (COH)	0.0001	0.0006

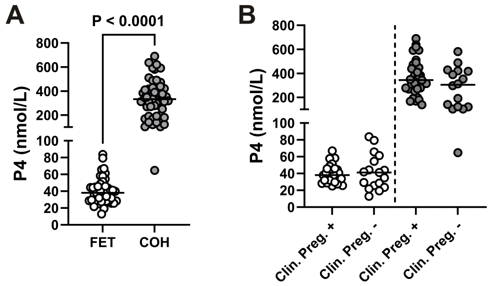

Supplementary Figure 4: Progesterone levels in women undergoing **A:** COH as compared to FET and **B:** women achieving pregnancy as compared to women not achieving pregnancy

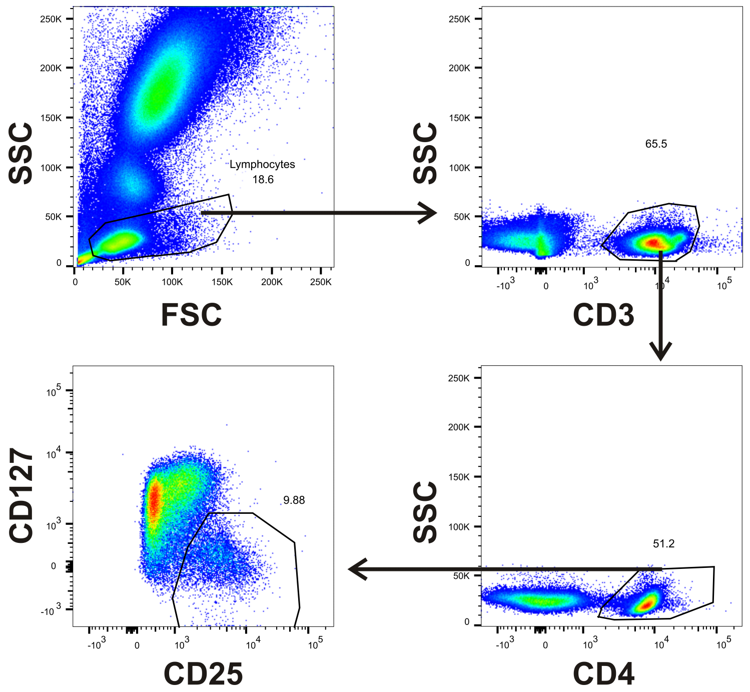

Supplementary Figure 5: Gating hierachy for the identification of CD25$^+$CD127$^{lo/-}$ Treg cells.

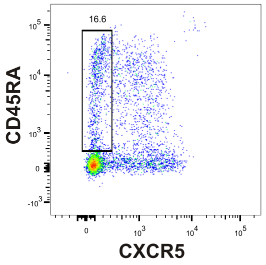

Supplementary Figure 6: Gating scheme for identification of the CXCR5-CD45RA+ nTreg subset among $CD25^{+}CD127^{lo/-}$ Treg cells is shown on the representative pseudocolor plot.
